# Society Meetings

**Published:** 1900-07

**Authors:** 


### SOCIETY MEETINGS.

The Lake Erie Medical Society held its regular quarterly meeting at Central Hotel, Angola, N.Y., June 29, 1900. The following program was announced: Diphtheria, E. W. Janes, Hamburg, N.Y.; Paper, C. S. Pugsley, Gowanda N. Y.; early and late operation for appendicitis, Eugene A. Smith, Buffalo, N.Y.; Treatment of retrodisplacements of the uterus, Herman E. Hayd, Buffalo, N.Y.

---

The Medical Society of the County of Chautauqua will hold its meeting at Chautauqua, July 10, 1900. Drs. Sidney Graber and W. S. Bainbridge, of New York City, and J. H. Pryor and Wm. C. Krauss, of Buffalo, N.Y., will read papers in the afternoon before the society.

---

The American Electro-therapeutic Association will hold its tenth annual meeting, September 25, 26 and 27, 1900, at the Academy of Medicine in New York City. Two discussions are announced as follows: Electricity in gynecology and the present reluctance of gynecologist to use electricity; Electricity in tuberculosis and the present modes of treatment.

The president is Dr. Walter H. White, 220 Marlborough Street, Boston, Mass.; Dr. Robert Newman, 148 West 73d Street, New York City, is chairman Committee on Arrangements; and Dr. Geo. E. Bill, 255 North Street, Harrisburg, Pa., is Secretary.

The Rochester Academy of Medicine held its regular quarterly meeting at the Reynolds Library, Wednesday, June 20, 1900. The surgical section provided the following program: I. Presentation of patients. (*a*) Tuberculosis of the wrist joint, E. W. Mulligan; (*b*) Abscess of the kidney, Henry T. Williams. II. Pathological specimens. (*a*) Fracture of the neck of the femur; (*b*) Hourglass constriction of the stomach; (*c*) Gallstone, Wm. L. Conklin. III. Discussion: Surgical tuberculosis. (*a*) Symptomatology and diagnosis of tubercular disease of the bones and joints, illustrated by lantern slides, radiographs and pathological specimens, Louis A. Weigel; (*b*) The surgical treatment of tuberculosis of the bones and joints, E. W. Mulligan; (*c*) Surgical treatment of tubercular peritonitis, J. P. Creveling, Auburn, N. Y. Dr. John O. Roe is the chairman, and Dr. E. Wood Ruggles, is the secretary.

---

The Buffalo Academy of Medicine held meetings during the month of June, 1900, as follows:

*Section on Surgery*—Tuesday evening, June 5th. Program: Bennett's New method of anesthesia, Prescott Le Breton; Some notes on gonorrheal rheumatism, with report of cases, J. Henry Dowd.

*Section on Ophthalmology, Otology, Rhinology and Laryngology.*—Monday evening, June 11th. Program: Contributing to the etiology and treatment of phlyctenular disease, Lorenzo Burrows; Further report of a case of tubercular tumor of the nasal septum, J. C. Clemesha; Effect of antitoxin serum on the blood, Geo. F. Cott.

*Annual Meeting.*—Tuesday evening, June 12, 1900. Program: Reports of officers. An address on Puerperal infection, by the retiring President, Matthew D. Mann. Discussion by Drs. P. W. Van Peyma, H. D. Ingraham, M. A. Crockett, L. G. Hanley and L. Schroeter.

*Section on Pathology.*—Tuesday evening, June 19th. Program: Unusual carcinoma of the colon and stomach, Herbert U. Williams. Discussed by Dr. Eugene A. Smith.

*Section on Obstetrics.* —Tuesday evening, June 26th. Program: Pelvic inflammation, M. A. Crockett; Nervous disturbances during menopause, Lillian C. Randall; Infant feeding, Maud J. Frye.

